# Supplementary material for: The Insertion Domain of Mti2 Facilitates the Association of Mitochondrial Initiation Factors with Mitoribosomes in Schizosaccharomyces pombe
Source: Biomolecules. 2025 May 10;15(5):695. doi: 10.3390/biom15050695 (PMC12109253; doi:10.3390/biom15050695)
Supplement: Supplementary file 1 [file biomolecules-15-00695-s001.zip › biomolecules-3605126-supplementary/biomolecules-3605126-supplementary.pdf]

Fig. S1

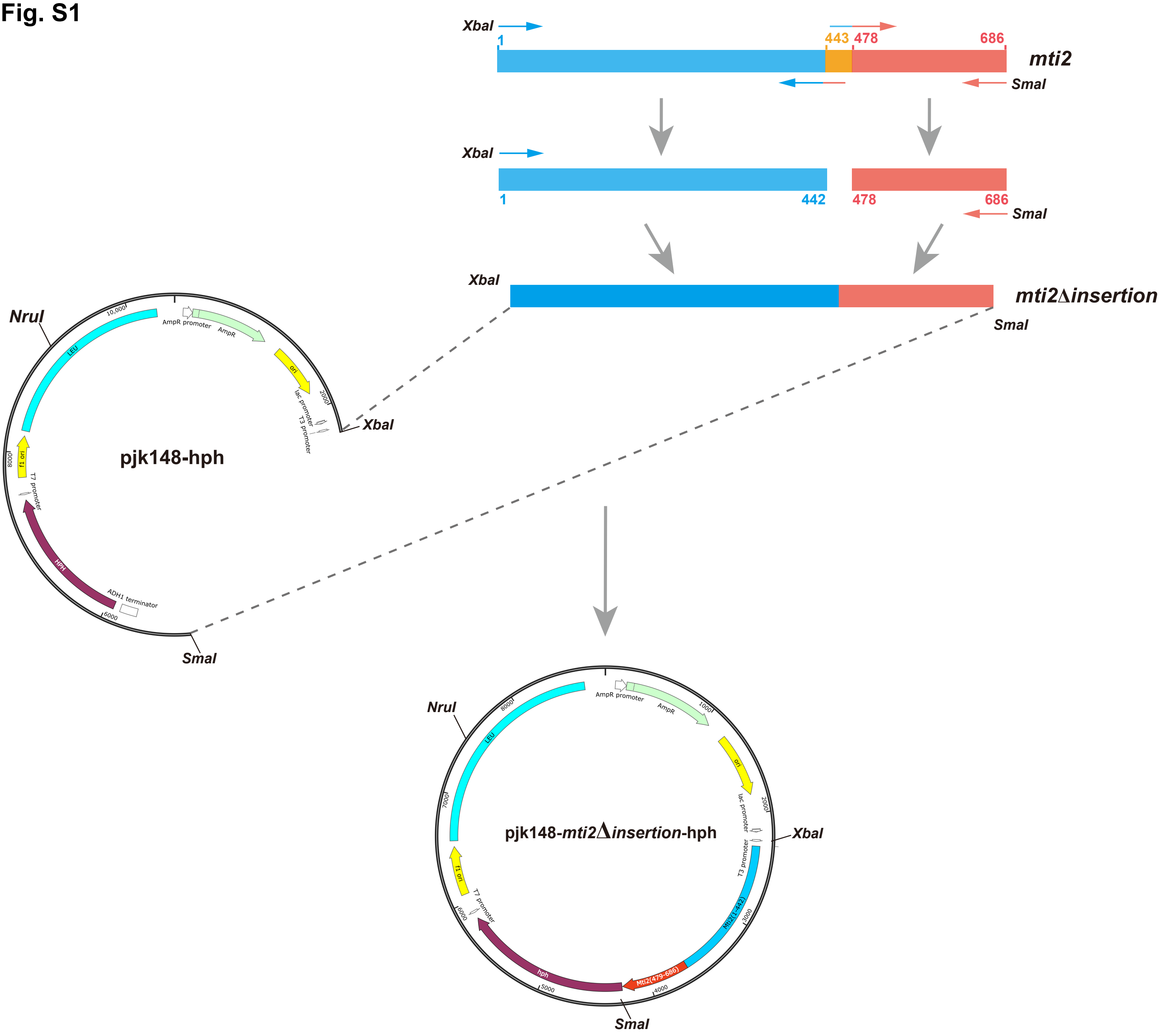

Fig. S1. The schematic view of the construction of *mti2Δinsertion* strain.

The amino acid regions 1-442 and 478-686 of *mti2* were amplified by PCR and subsequently fused into a single fragment by overlapping PCR. The resulting PCR product was cloned into XbaI/SmaI sites of the pjk148-hph plasmid, generating the *mti2Δinsertion*-hph deletion cassette construct. This cassette was subsequently transformed into wild-type strain to generate *mti2Δinsertion* mutant strain.

Fig. S2

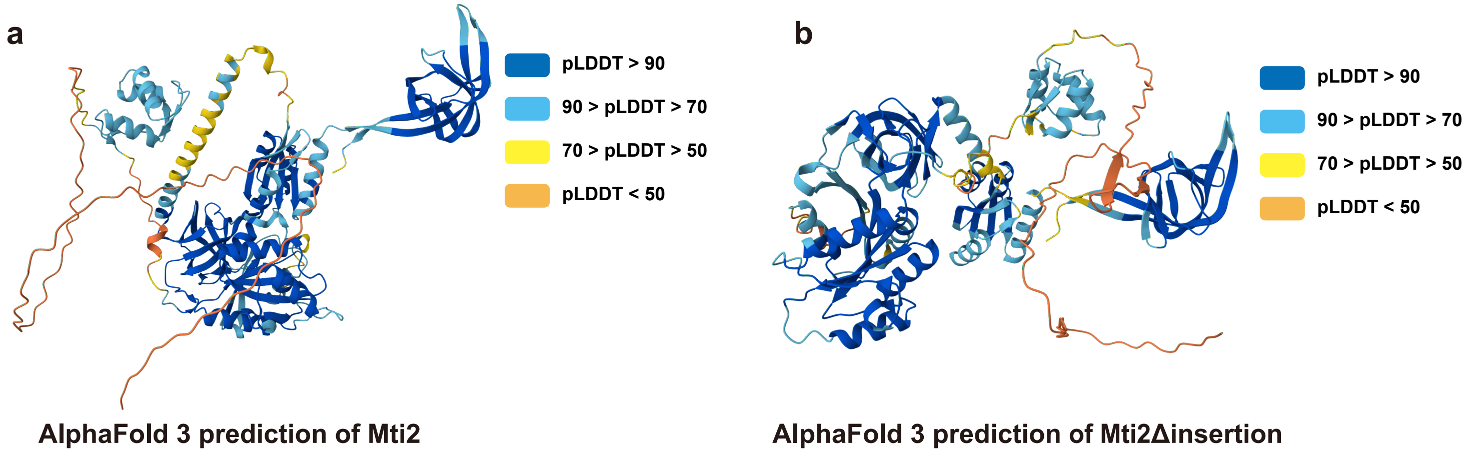

Fig. S2. AlphaFold 3 predictions of Mti2 and Mti2Δinsertion colored by pLDDT confidence scores. AlphaFold 3 predictions of full-length Mti2 (**a**) and Mti2Δinsertion (**b**) are colored according to the pLDDT confidence scores. Dark blue (pLDDT > 90) indicates very high confidence; light blue (90 > pLDDT > 70) indicates confident prediction, yellow (70 > pLDDT > 50) indicates low confidence, and orange (pLDDT < 50) indicates very low confidence.

Table S1 List of *S. pombe* strains used in this study

Strain	Genotype	Source
yHL6381	*h- leu1-32 his3-D1 ura4-D18 ade6-M210*	Lab stock
yLY1	*h- leu1-32 his3-D1 ura4-D18 ade6-M210 mti2:: kanMX6*	This study
yLY2	*h- leu1-32 his3-D1 ura4-D18 ade6-M210 mti2:: [mti2*ΔInsertion- *hphMX6]*	This study
yLY3	h^- *leu1-32 his3-D1 ura4-D18 ade6-M210 mti2*:: *mti2-FLAG-hphMX6*	This study

Table S2 List of primers used in this study

Primer names	Primer sequences (5′ to 3′)	
Primers for the deletion of *mti2*		
Δ*mti2*-up-F	ACGCGTCGACGAAAGAGACAATCGTTAGTGTG	
Δ*mti2*-up-R	GGAAGATCTTTTAGGAAATGAATTAAACGTG	
Δ*mti2*-down-F	CGGAATTCCATGCACTTCTATTTAATCCTGG	
Δ*mti2*-down-R	TCCCCGCGGACGGAACTGAACCATTGAAG	
Check primers for the verification of *mti2* deletion		
Δ*mti2*-yz-up-F	TATACGAACGACTTGATGAGC	GGACAATTCAACGCGTC
Δ*mti2*-yz-kanMX6-R	GGACAATTCAACGCGTC	GCCTGTTGAACAAGTCTG
Δ*mti2*-yz-kanMX6-F	GCCTGTTGAACAAGTCTG	GAACAATGGGTATAGACTCTG
Δ*mti2*-yz-down-R	ATTAGTAACTCTGCATTGGTG	GACAGCATAGTAAACGGC
Primers for the deletion of insertion domain of *mti2*		
Mti2$^{1-442}$-F-XbaI	GCTCTAGAATGGCTTTTTGTCTGCATG	
Mti2$^{1-442}$-R	TAAAGGATTTAGATACCTGACGATATATATCAGAAAGGATACG	
Mti2$^{478-686}$-F	TCTGATATATATCGTCAGGTATCTAAATCCTTTAATATCATTGC	
Mti2$^{478-686}$-R-SmaI	TCCCCCGGGCTAAAAATCAGGAGGTTTGTATTCC	
Primers for strain expressing Mti2-FLAG		
Mti2-FLAG-up-F	CGAGTCTTGTATACTGGGGTGG	
Mti2-FLAG-up-R	TCCTTGTAGTCAAAATCAGGAGGTTTGTATTC	
Mti2-FLAG-hphMX6-F	CTGATTTTGACTACAAGGACGACGATG	
Mti2-FLAG-hphMX6-R	GAAGTGCATGCTATTCCTTTGCCCTC	
Mti2-FLAG-down-F	GGCAAAGGAATAGCATGCACTTCTTATTTAATCC	
Mti2-FLAG-down-R	CGATGAGACCCGTGCTAGC	
Primers for qRT-PCR		
actin-RT-F	TCCGCTCTTAACATCTCATGAGG	
actin-RT-R	AAGGCTAGCTCTGCATTCGTCTAT	
cob1-RT-F	GCCTTTTGTTATTGCTGCTTTA	
cob1-RT-R	GTTATCAAATCTTTTATCAGATAAT	
cox1-RT-F	TGGACGGTATATCCACCACT	
cox1-RT-R	GTCGCTATTAAATTTACTGATCC	
cox2-RT-F	AAGTGGTGATGTTATCCATAGTTGG	
cox2-RT-R	AGATACACCTTGAACAACAATAGGC	
cox3-RT-F	CCACCAGTAGGAATAGCAGATAAAA	
cox3-RT-R	TGAGCATAAGTTAAACTAGCACCAG	
atp6-RT-F	TACCTTCTGGTACTCCTACTCC	
atp6-RT-R	TAGCACCTAATCGAATACCTAAACTT	
atp8-RT-F	ATGCCACAATTAGTACCATTCT	
atp8-RT-R	AAAGAACTTATAATAGATCTTGAG	
atp9-RT-F	GGTGCTGGTGTTGGTATTGGA	
atp9-RT-R	ACCTGTAGCTTCTGTTAAGGCG	
rns-RT-F	GAAGGAGGAATTGCGAGTAATCAC	
rns-RT-R	CGACTTAACACTAATTGCACAACACC	
rnl-RT-F	GTAGCACGGTAGTAAAGCCAAATTG	
rnl-RT-R	TAAGGATTTGTACATCCTAAGGATGTCC	
var1-RT-F	AGAGCTCTTCCTATTTCAACTCCTT	
var1-RT-R	ACCTTTCCATCCTTTTGGTACA	

File S1: Original western blotting figures

Fig. 2d

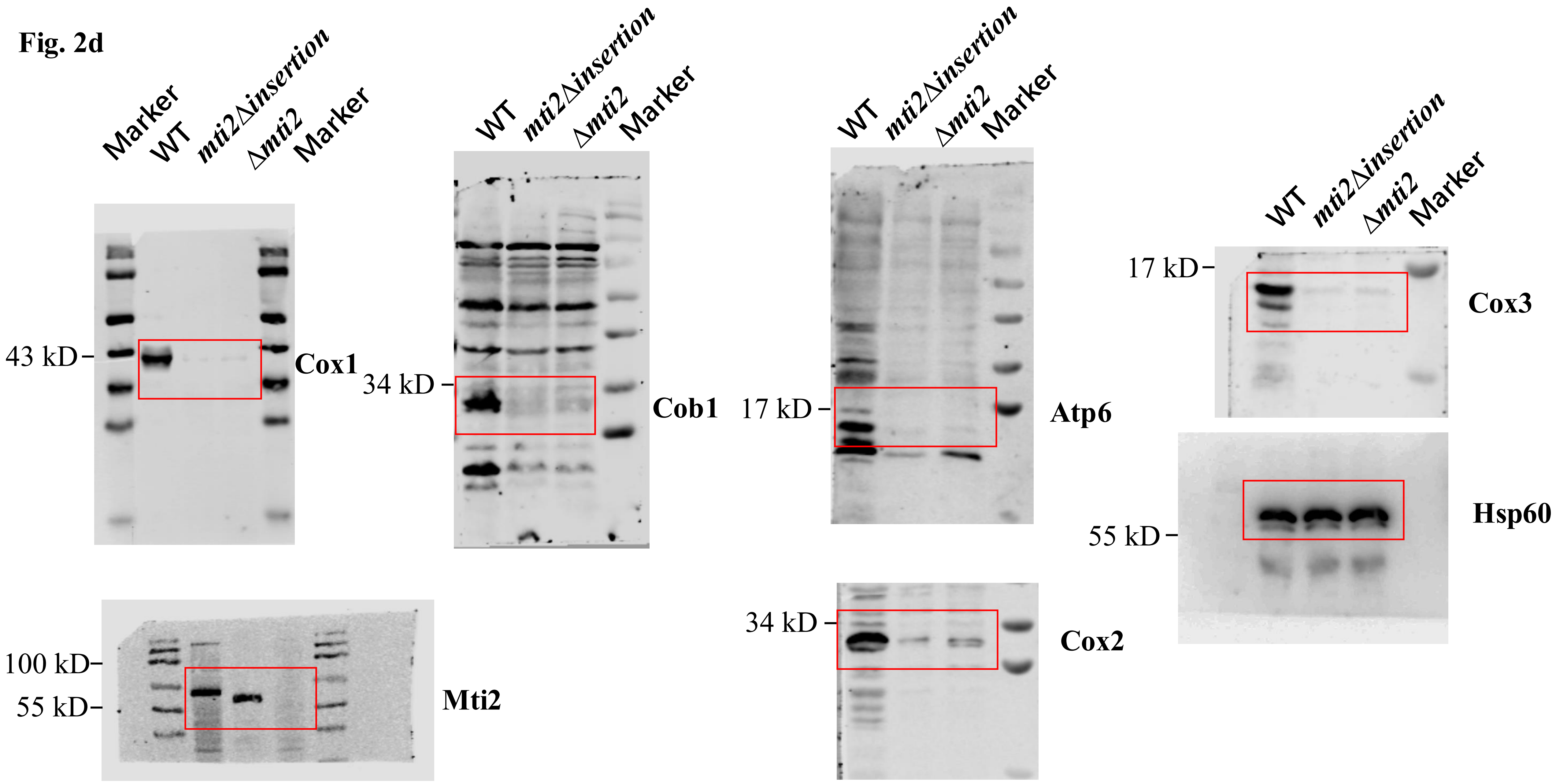

Fig. 3

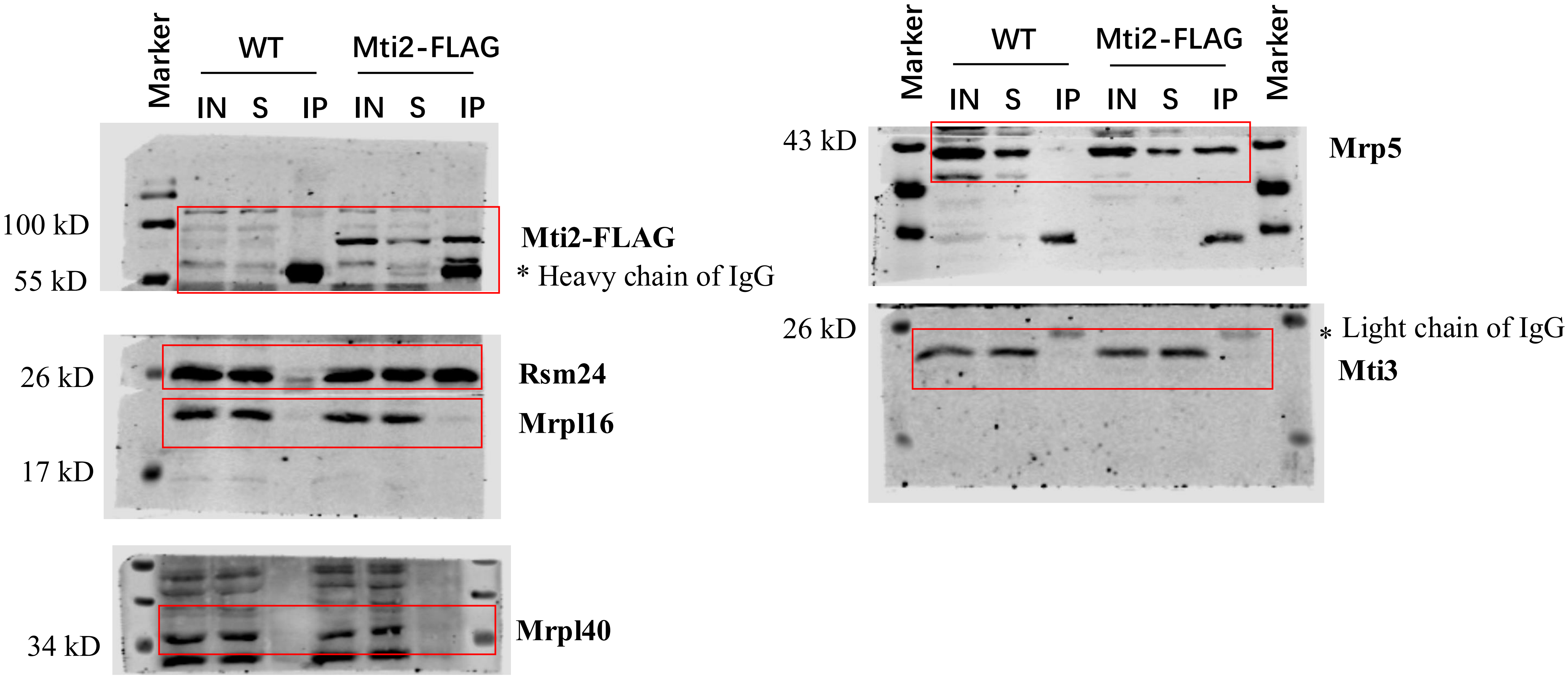

Marker
WT
Mti2-FLAG
IN
S
IP
IN
S
IP
100 kD
55 kD
Mti2-FLAG
* Heavy chain of IgG
26 kD
17 kD
Rsm24
Mrpl16
34 kD
Mrpl40
Marker
WT
Mti2-FLAG
Marker
IN
S
IP
IN
S
IP
43 kD
Mrp5
26 kD
* Light chain of IgG
Mti3

Fig. 4a

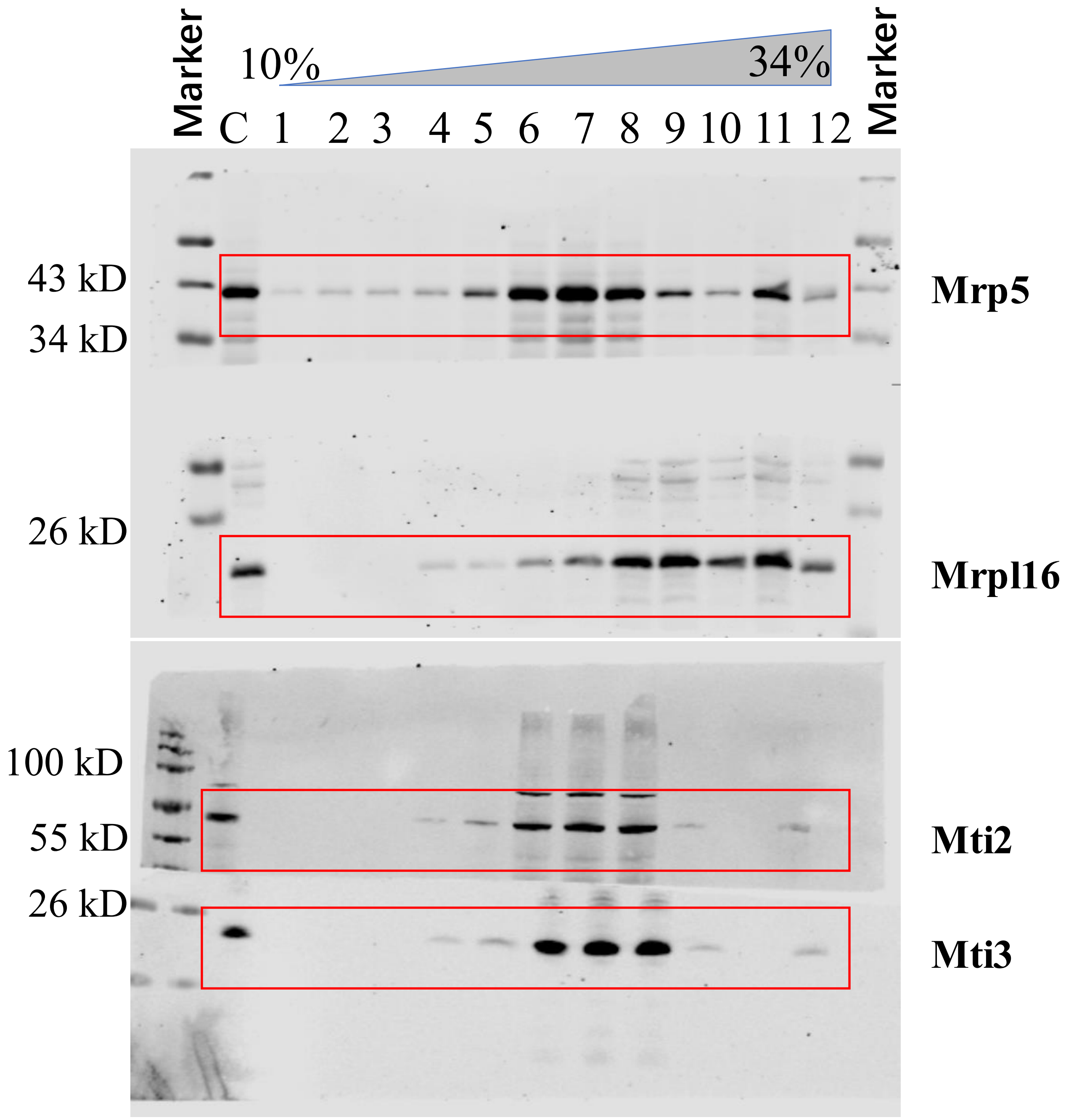

Marker
10%
34%
C 1 2 3 4 5 6 7 8 9 10 11 12
Marker
43 kD
34 kD
Mrp5
26 kD
Mrpl16
100 kD
55 kD
Mti2
26 kD
Mti3

Fig. 4b

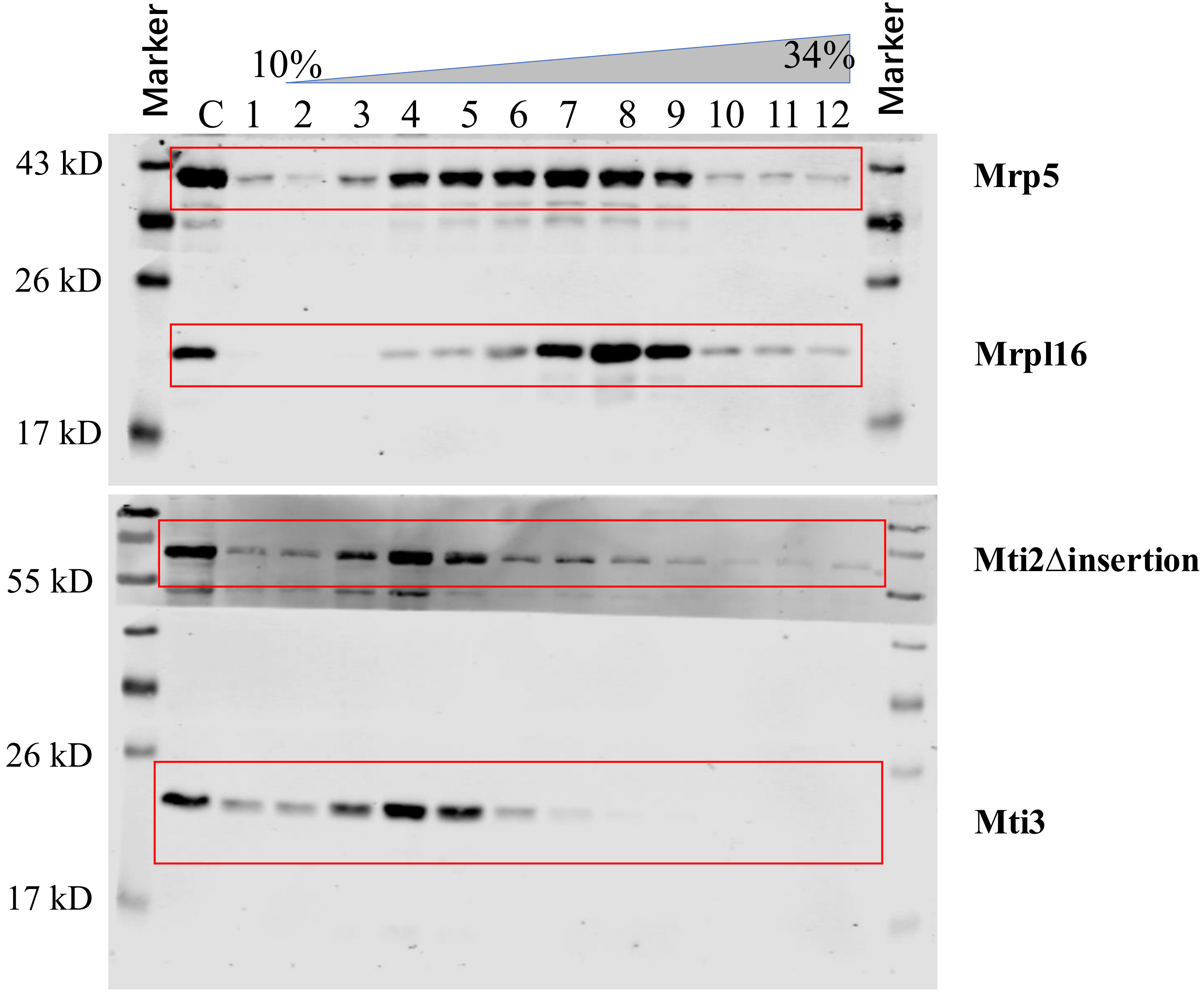

Marker
C 1 2 3 4 5 6 7 8 9 10 11 12
10%
34%
Marker
43 kD
26 kD
17 kD
Mrp5
Mrpl16
55 kD
26 kD
17 kD
Mti2Δinsertion
Mti3

Fig. 4c

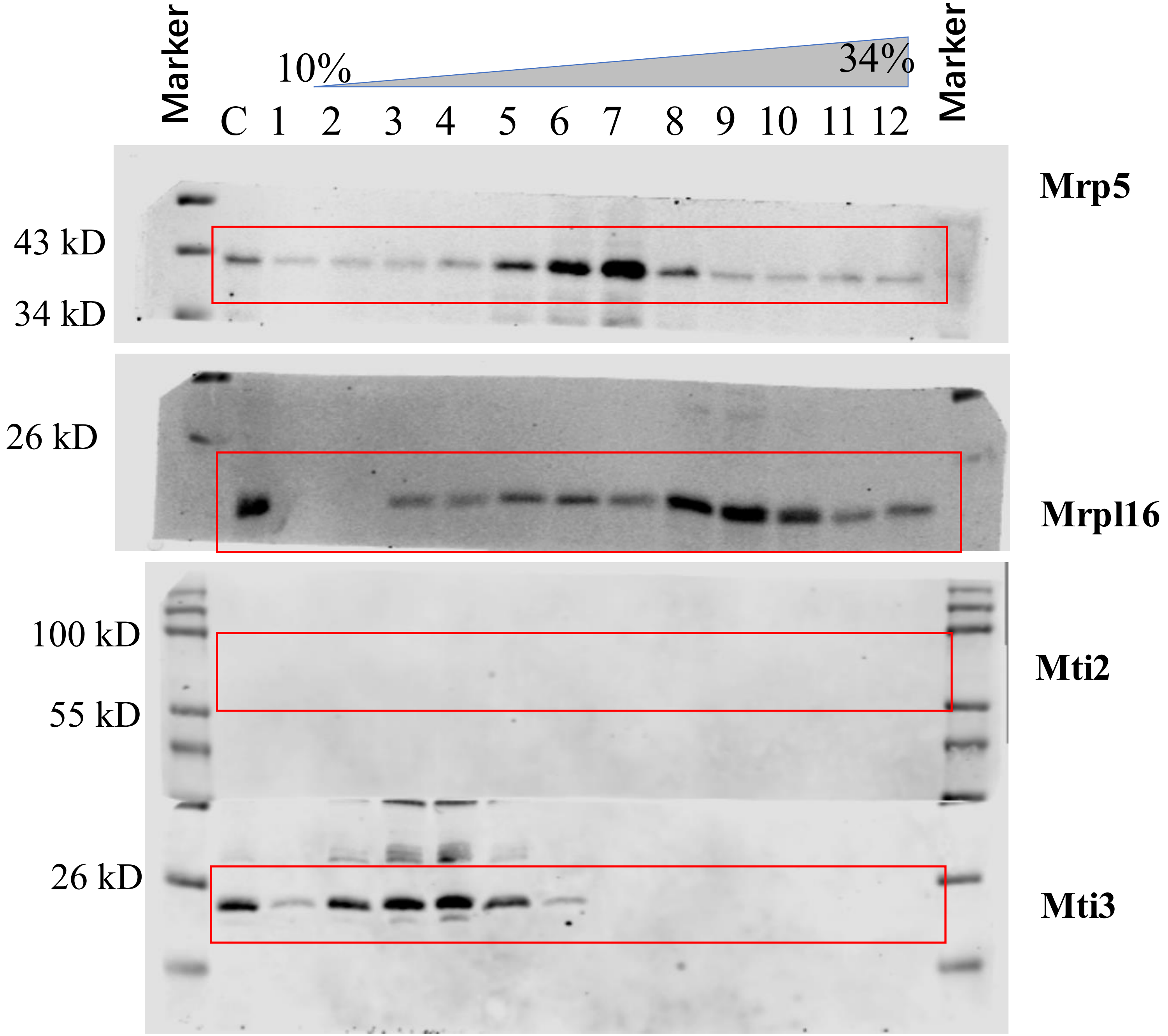

Marker
10%
34%
Marker
C 1 2 3 4 5 6 7 8 9 10 11 12
43 kD
34 kD
Mrp5
26 kD
Mrpl16
100 kD
55 kD
Mti2
26 kD
Mti3
