# Supplementary figures and images for: The Insertion Domain of Mti2 Facilitates the Association of Mitochondrial Initiation Factors with Mitoribosomes in Schizosaccharomyces pombe
Source: Biomolecules. 2025 May 10;15(5):695. doi: 10.3390/biom15050695 (PMC12109253; doi:10.3390/biom15050695)

Fig. 2d

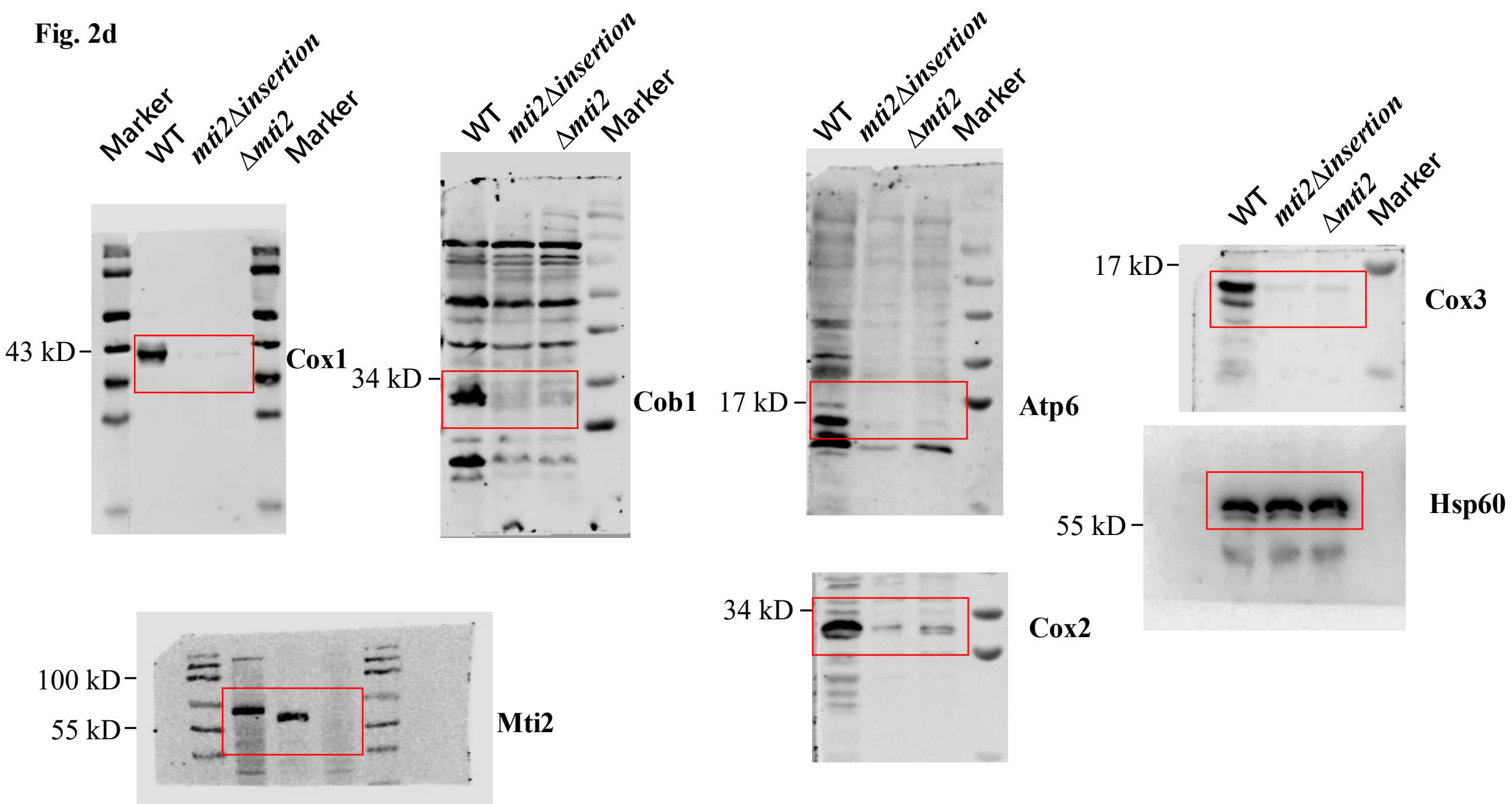

Fig. 3

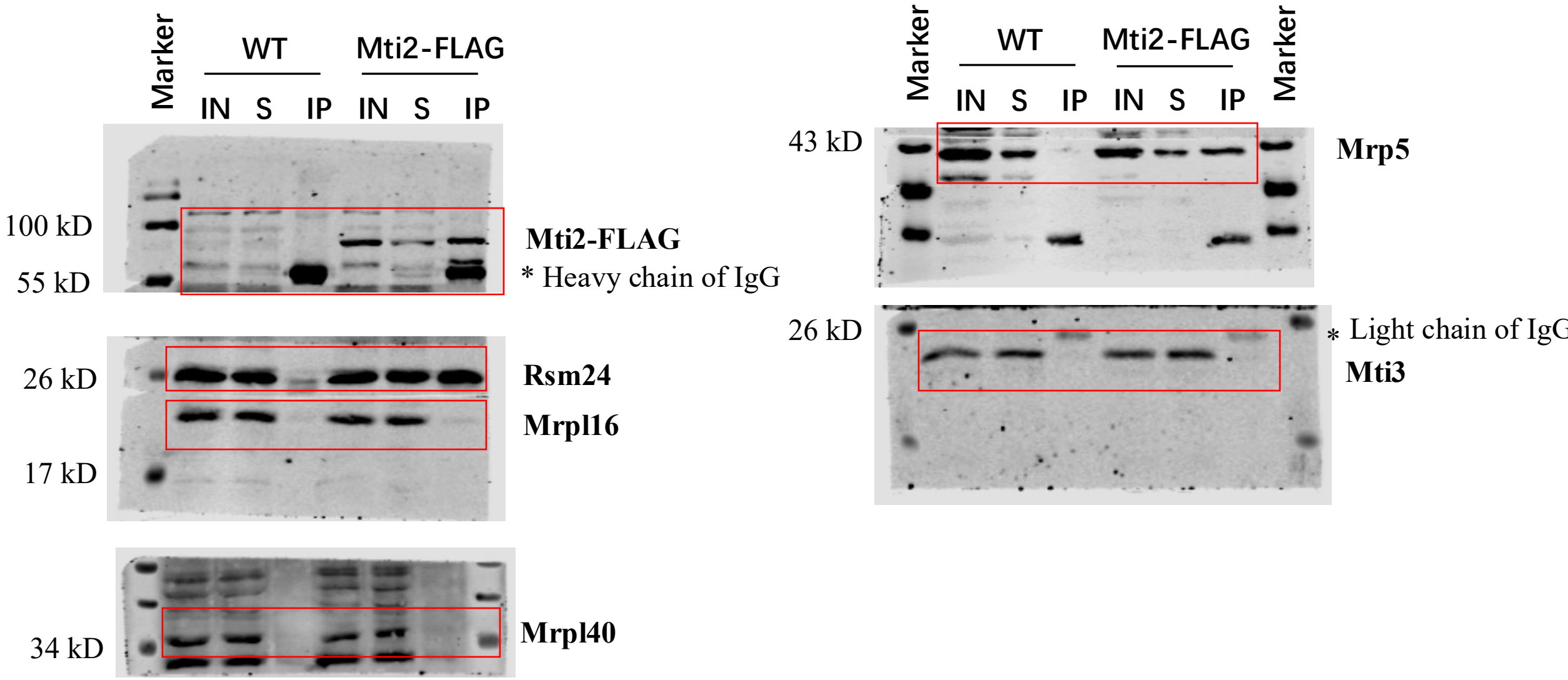

**Fig. 4a**

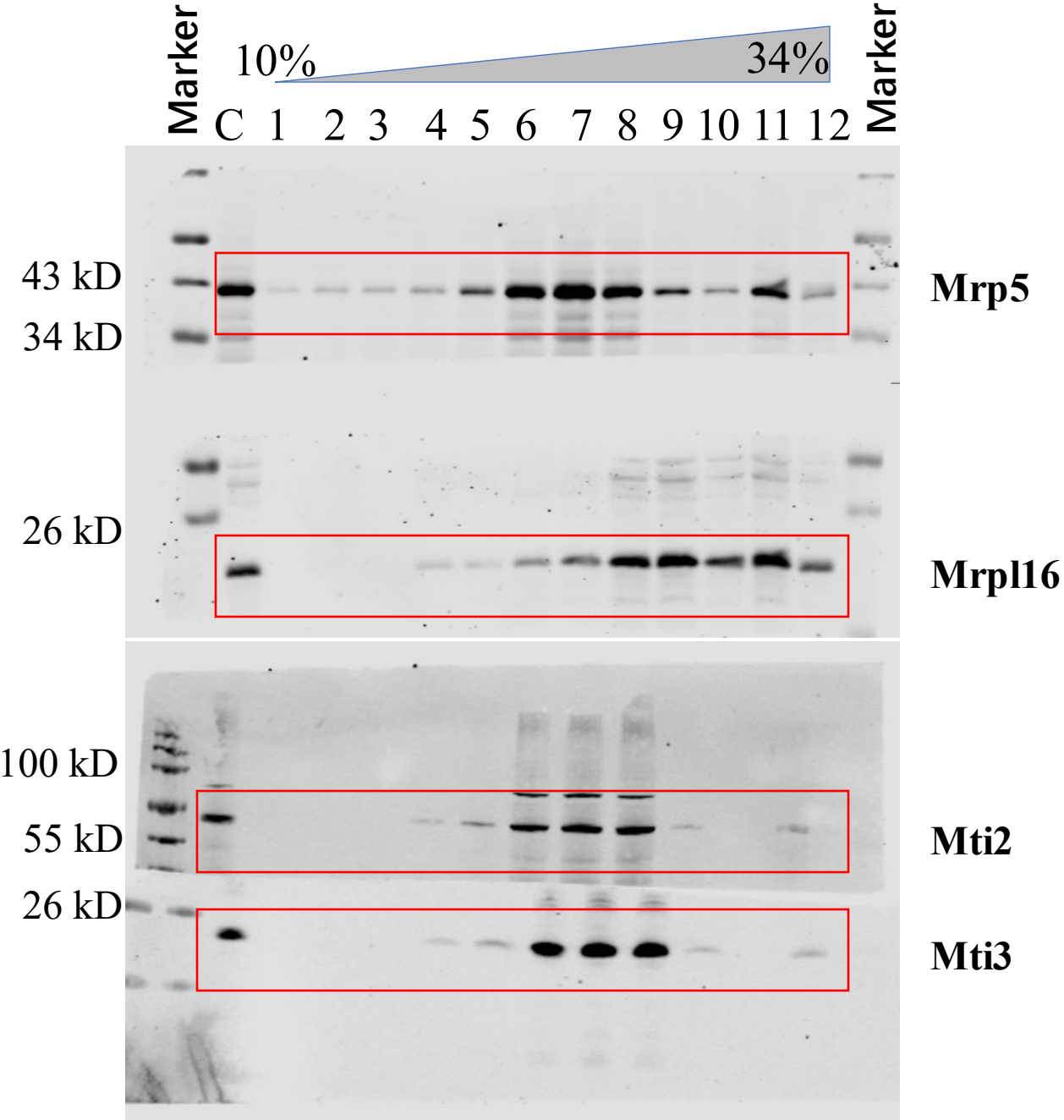

Fig. 4b

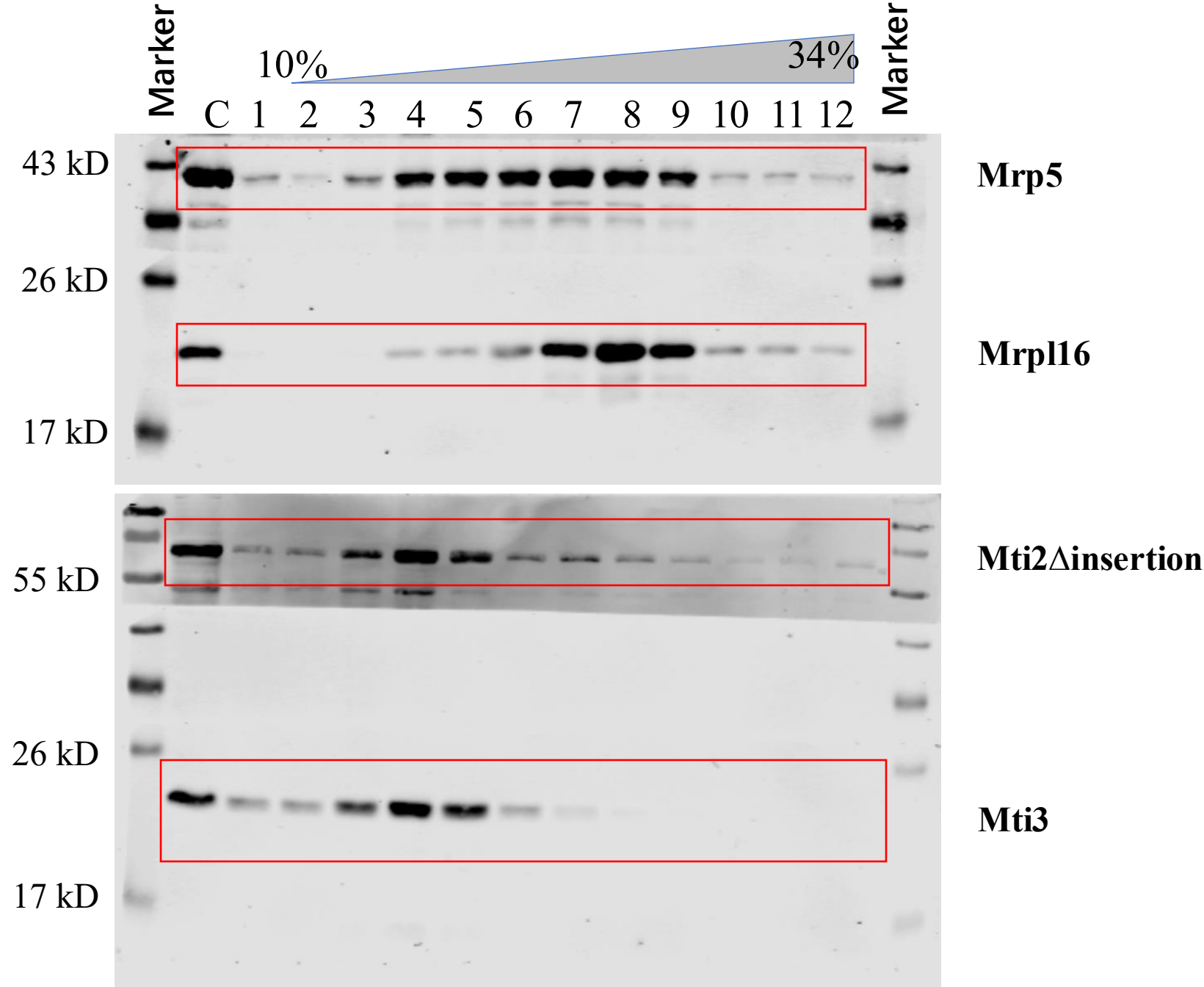

**Fig. 4c**

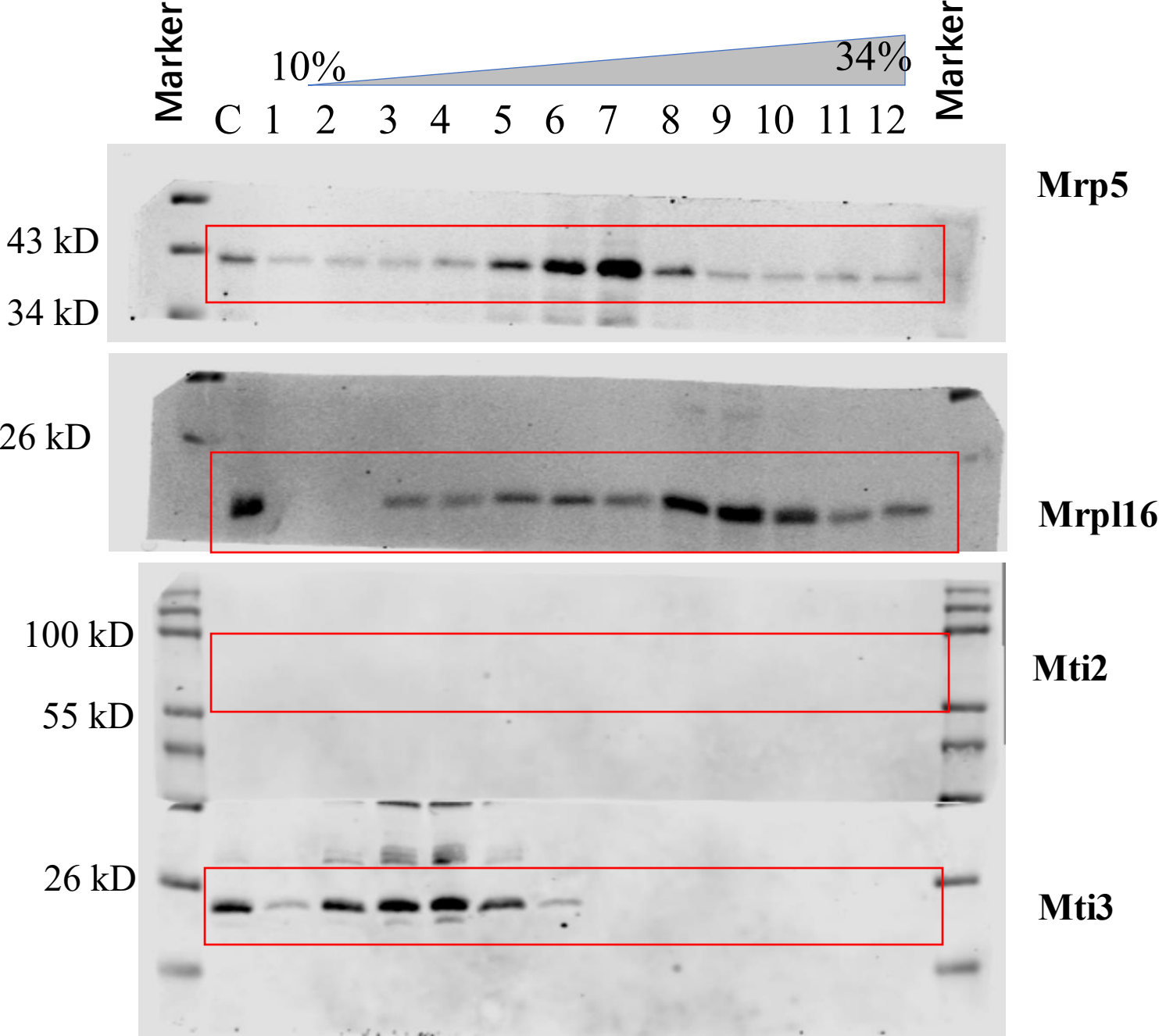

Supplement: Supplementary file 1 [file biomolecules-15-00695-s001.zip › biomolecules-3605126-supplementary/Updated Supplementary File S1 Original western blotting figures.pdf]
